# Researches and applications of pollution prevention and control technologies for carbon dross from electrolytic aluminium production: a review

**DOI:** 10.1039/d5ra04272k

**Published:** 2025-10-16

**Authors:** Ningning Feng, Chenquan Wang, Chunqiang Chen, Xi Liu, Qiang Huo

**Affiliations:** a School of Life Science and Technology, Inner Mongolia University of Science & Technology Baotou 014010 China; b Key Laboratory of Ecology of Rare and Endangered Species and Environmental Protection (Guangxi Normal University), Ministry of Education Guilin Guangxi 541006 China huoqiang@gxnu.edu.cn; c Jiading District Environmental Monitoring Station Shanghai 201822 China

## Abstract

This review focuses on the long-overlooked carbon dross from aluminum electrolysis, a hazardous waste enriched in carbon and high-fluoride salts, despite its relatively low generation volume. With the continuous expansion of global aluminum production, the output of carbon dross has increased accordingly, yet its environmental risks and resource potential have not received sufficient attention. For the first time, this article provides an examination of carbon dross following a “generation-hazards–prevention-control” framework. It elucidates the formation mechanism *via* corrosion, spalling, and entrainment of carbon anodes in electrolytic cells, and analyzes the release behavior and ecotoxicological effects of its toxic components. The limitations of existing end-of-pipe treatment technologies are critically assessed. Furthermore, based on the 3C (clean-cycle-control) green development strategy, a management framework is proposed: clean first, through the adoption of inert anodes, energy-efficient electrolysis processes, and intelligent optimization to minimize dross generation at the source; for unavoidable dross, the cycle is employed to achieve high-value recovery of aluminum fluoride and functional carbon materials; finally, by integrating cross-scale environmental risk assessment and policy instruments, a science-technology-management integrated control decision-making system is established, offering a paradigm for low-carbon, high-value, and safe management of carbon dross.

## Abstract 2

Based on the 3C (Clean–Cycle–Control) strategy, this study reviews progress in source reduction, process recycling, and end-of-pipe treatment of aluminum electrolysis carbon dross, offering a paradigm for its low-carbon, high-value, and safe management.

## Graphical abstract

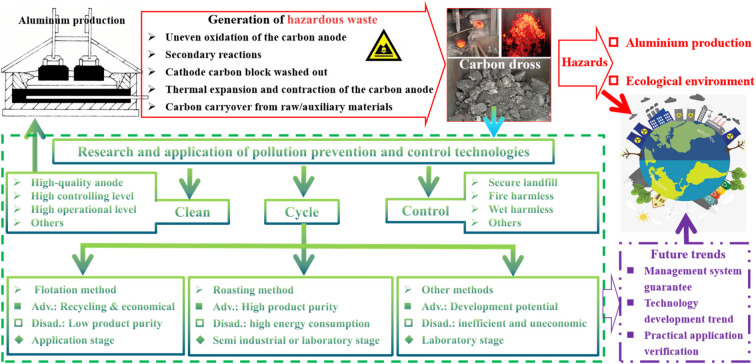

## 1. Introduction

**Fig. 1:**
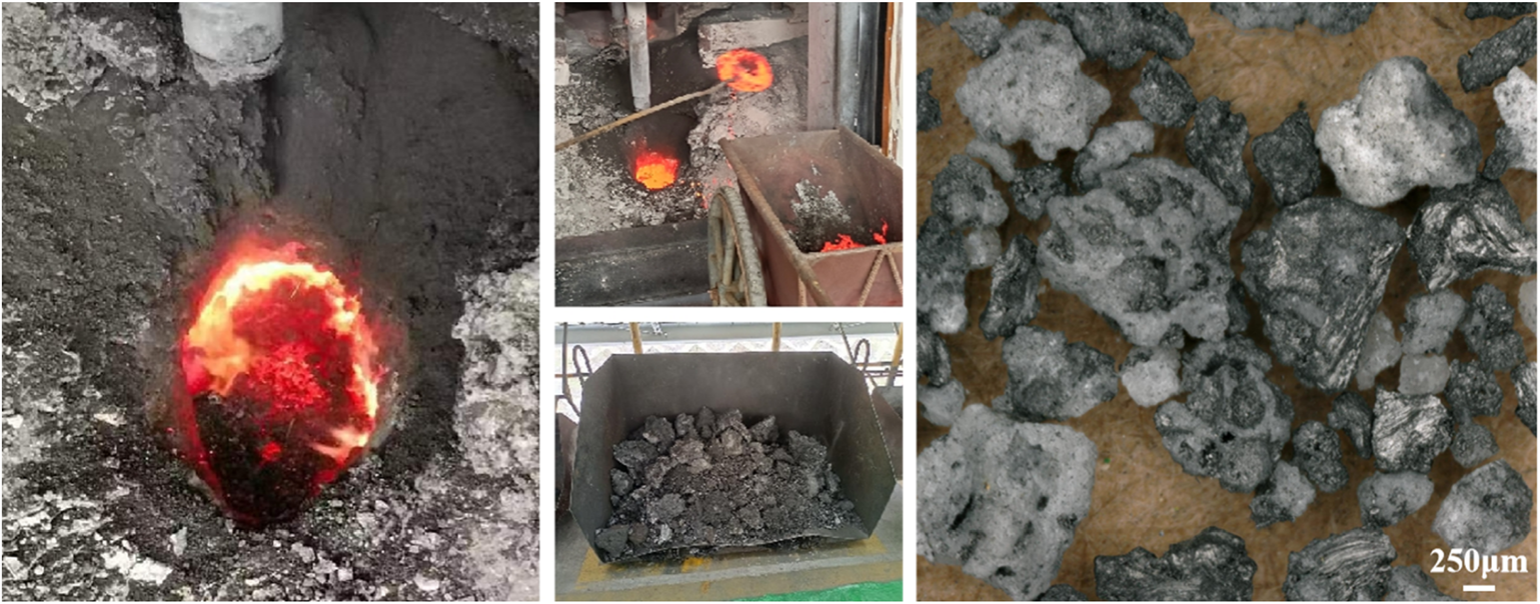
Removal, temporary storage, and microstructural characterization of carbon dross in an aluminum electrolysis plant in China.

Aluminum is produced through the Hall–Heroult electrolysis process.^1,2^ During the aluminium electrolysis process, the surface of the electrolyte melt becomes contaminated with floating objects resulting from carbon anode slags, cathode carbon block slags and other materials. The floating objects adversely affect the technical and economic indicators of the production process and must be promptly removed from the electrolytic cell. Consequently, carbon dross, a by-product of aluminium electrolysis,^3^ which is also called carbon-anode residue, spent carbon anode or carbon dust, is generated (Fig. 1).

**Fig. 2:**
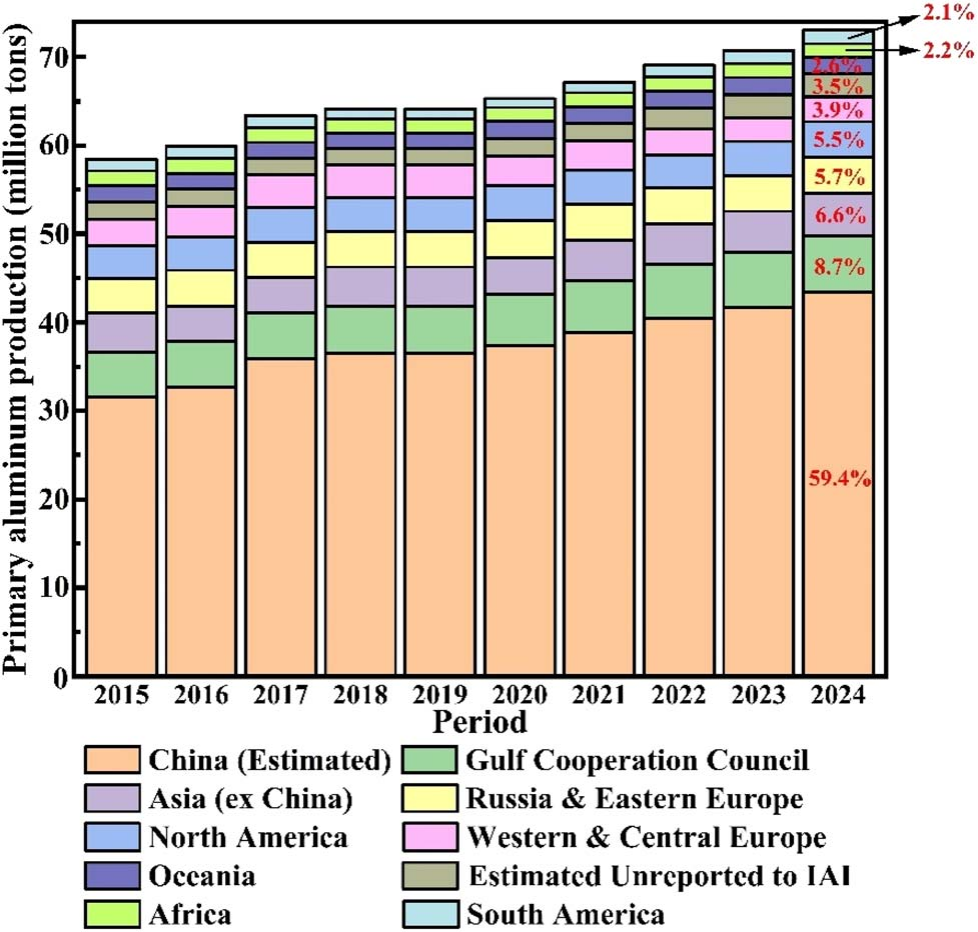
Global primary aluminum production from 2015 to 2024 (note: the image data is derived from public data provided by the IAI).

Aluminum is the second most demanded metal globally after steel.^4^ According to statistics from the International Aluminium Institute (IAI) between 2015 and 2024 (Fig. 2, available at https://international-aluminium.org), the global primary aluminum production is highly concentrated in specific regions, with China being the dominant producer of electrolytic aluminum. Over the past decade, China's share in the global production has continued to grow, reaching 43.396 million metric tons in 2024, which is 59.4% of the world's total output (73.009 million metric tons).^5^ Production statistics reveal that the production of each ton of primary aluminium generates approximately 5 to 15 kg of carbon dross; individual enterprises using poor-quality carbon anodes can produce up to 50 kg of carbon dross.^6,7^ Even with a median production rate of 10 kg per ton of aluminum, more than 700 000 tons of carbon dross will be produced globally each year.

The primary constituents of carbon dross are carbon and electrolytes such as Na_3_AlF_6_, NaF, AlF_3_, CaF_2_, KF, and LiF.^2^ Improper disposed of carbon dross can lead to fluoride ions (F^−^) entering the atmosphere, water bodies, and soil through dust, leachate, and other processes, thereby contaminating the ecological environment and posing risks to the human health and safety.^8^ The United States Environmental Protection Agency (EPA) classifies waste generated during primary aluminum production as a K-listed hazardous waste and includes it in the hazardous waste management inventory regulated under the Resource Conservation and Recovery Act (RCRA).^9^ In the European Union, the Waste Framework Directive (2008/98/EC) and the European Waste Catalogue (EWC) explicitly identify waste from aluminum smelting processes as highly flammable and hazardous.^10^ In 2016, China's National Catalogue of Hazardous Wastes included salt residue and floating dross generated during the electrolytic aluminum production process under hazardous waste regulation, but the classification criteria were vague.^11^ The revised 2021 version clearly defined carbon dross produced in the electrolytic aluminum process as hazardous waste.^12^ “Carbon dross produced in the process of aluminium electrolysis” belongs to “HW48 nonferrous metal mining and smelting waste,” with the hazardous waste code 321-025-48 and the hazardous characteristics of Toxicity (T). China's environmental protection departments have established varying fees for the disposal of industrial hazardous waste based on local circumstances, typically ranging from RMB 800 to 2000 per ton. According to the Environmental Protection Tax Law of the People's Republic of China, which has been in effect since 2016, each ton of hazardous waste is subject to an additional environmental protection tax of RMB 1000.^13^ Consequently, the external disposal cost of carbon dross has emerged as a significant expense in the operation of aluminium-manufacturing enterprises.

Under the premise of the global aluminum industry's transformation towards green recycling and low-carbon emissions, carbon dross, which was only recently classified as hazardous waste, still receives insufficient attention. To advocate for more researchers to focus on the pollution prevention and control of carbon dross, this study investigates the formation mechanisms and potential hazards of carbon dross and reviews cleaner production technologies for reducing its generation. In addition, recycling strategies are summarized. Based on these findings, we propose recommendations for pollution prevention and control to enable safe and efficient hazardous-waste management and facilitate the development of a green, high-efficiency, and sustainable electrolytic aluminum industry.

## 2. Carbon dross generation and hazards

### 2.1. Generation of carbon dross

**Fig. 3:**
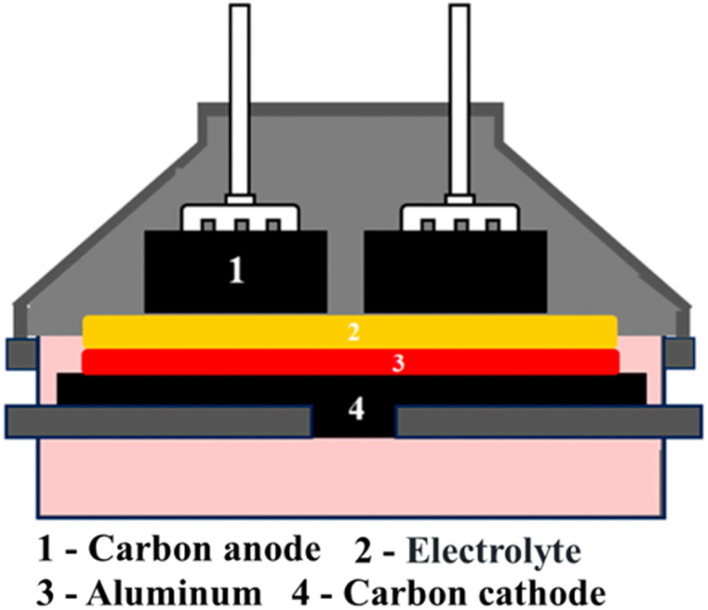
Structure of an aluminum electrolysis cell.^14^

Carbon dross forms mainly from uneven anode oxidation, thermal cycling, and secondary reactions inside the aluminum electrolysis cell (Fig. 3),^14^ as well as cathode spalling and external carbon input.

The net electrochemical reaction of the aluminum electrolysis cell inside the pot is expressed as follows:^15^a
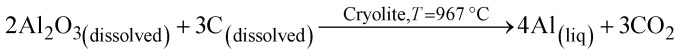

b At cathode: 4Al_(bath)_^3+^ + 12*e*^−^ → 4Al_(liq)_

c At anode: 3C_(dissolved)_ + 6O_(aq)_^2−^ → 3CO_2(g)_ + 12*e*^−^

Thermodynamic analysis of the electrochemical reactions in the aluminum electrolysis cell (Text S1 in SI).

#### 2.1.1. Uneven oxidation of the carbon anode

##### 2.1.1.1 Uneven oxidation mechanism

The carbon anode consists mainly of aggregates and binders, with the aggregate composed of less reactive petroleum coke particles and the binder containing the more reactive bitumen. The reaction in eqn (a) preferentially consumes the more reactive binder, and the less reactive aggregate,^16^ which reacts fully at a lower rate than the more reactive binder, is shed into the electrolytes and forms carbon dross.^17^

##### 2.1.1.2 Quality of carbon anode

The carbon anode quality is a key factor influencing uneven oxidation, determined by raw materials and production processes.^18^ Variations in equipment, management, process control, and cost can lead to quality defects, such as unsynchronized oxidation of aggregates and binder,^19^ high air permeability or low air reactive residual rate. Trace elements (such as sodium, vanadium, and nickel) present in petroleum coke or asphalt reduce oxidation resistance. Sodium, in particular, acts as a catalyst. During the aluminium electrolytic process, the geometry of the carbon anode affects the electromagnetic, fluid and temperature fields, concentrating stress in certain areas and resulting in increased carbon dross formation.^20^

##### 2.1.1.3 Aluminium electrolysis operation level

The operational precision of aluminium electrolysis greatly affects the carbon dross generation. Placing a new anode too low inhibits full-current operation and causes slag formation through electrolyte interaction.^21^ Even with correct placement, small anode spacing can cause thermal stress and anode bursting at full current, leading to dross from electrolyte scouring.^18^ Incomplete sealing after replacement or damaged insulation exposes the anode to air, resulting in oxidation and carbon dross.^22,23^ Inadequate thickness or an uneven size of the anode cover material reduces protection, exposing corners near outlets and flues to air and increasing oxidation. Poor preheating of salvage tools cools and solidifies electrolytes, which are removed with the dross, increasing the measured output. Worker skill in salvage operations also influences the final carbon dross yield.

#### 2.1.2. Secondary reactions

##### 2.1.2.1 Secondary reaction mechanism

Aluminium dissolved in the electrolyte solution during electrolytic aluminium production reacts with the anode gases CO_2_ and CO to produce free fine-grained carbon dross, as follows:^22^

d 2Al_(dissolved)_ + 3CO_(g)_ → Al_2_O_3_ + 3C_(s)_

e 2Al_(dissolved)_ + 3CO_2(g)_ → Al_2_O_3_ + 3CO_(g)_

f 4Al_(dissolved)_ + 3CO_2(g)_ → 2Al_2_O_3_ + 3C_(s)_

##### 2.1.2.2 Causes of generation

Certain specific production process conditions, such as an abnormal voltage, a high molecular ratio of electrolytes, an elevated electrolytic temperature, and an abnormal pole pitch, can lead to decreases in the current efficiency, increases in secondary reactions, and loss of aluminium while generating carbon dross. Among these secondary reactions, eqn (f) plays a more significant role in slag generation compared to eqn (d) and (e). Nevertheless, this secondary reaction is not the most important cause of carbon dross generation.

#### 2.1.3 Cathode carbon block washed out

During aluminium electrolysis, sodium and vanadium enter the electrolyte solution along with alumina. Due to contact between the electrolyte solution and the cathode carbon block, these impurities erode and penetrate the carbon block, causing it to expand in volume. This expansion can lead to cracking, loosening and porosity within the cathode carbon block. Under the washing action of molten electrolytes, slag is generated and floats on the surface of the electrolytes. The proportion of slag produced by the cathode carbon block is approximately 1–10% of the total amount of carbon dross.

#### 2.1.4. Thermal expansion and contraction of the carbon anode

The carbon anode is initially maintained at room temperature. When immersed in the molten electrolytes (approximately 950 °C), the violent temperature change causes violent expansion within a certain depth of the carbon material on the anode contact surface. The electrolyte always flows under the action of an electromagnetic field. The flow of the electrolyte produces a strong scouring force on the cracked coke aggregate and causes the aggregate to fall off from the anode carbon body, which forms a large amount of carbon dross in the electrolytes. This is one of the main sources for the mass production of carbon dross in the electrolyte, particularly at the beginning of the anode-replacement process.

#### 2.1.5. Carbon carryover from raw/auxiliary materials

##### 2.1.5.1 Alumina carryover

Before feeding into the electrolytic cell, alumina serves as an adsorbent to capture impurities, such as fluorine and carbon, in the electrolytic flue gas. On the other hand, alumina retains traces of carbon even after roasting. These two parts of carbon are brought into the electrolytic cell by alumina and become contributors to the generation of carbon dross.

##### 2.1.5.2 Anode-covering material carryover

The anode cover material above the molten electrolytes, which typically contains 2–5% carbon, serves as insulation for the electrolytic cell. A fraction of these materials infiltrates the electrolyte system during the electrolysis process. Carbon components that resist melting contribute to the formation of carbon dross.

### 2.2. Hazards of carbon dross

The accumulation of a significant amount of carbon dross in the electrolytic cell can have a detrimental impact on the aluminum production process, as well as on its technical and economic performance. Therefore, it is essential to periodically remove the carbon dross from the electrolytic cell. However, if the salvaged dross is disposed of haphazardly, the large amount of soluble fluoride contained in carbon dross may pose a significant threat to the ecological environment.

#### 2.2.1. Hazards to the electrolytic aluminium production process

##### 2.2.1.1 Disruption of the electrolytic aluminium production process

The presence of carbon dross poses significant disruptions to the electrolytic aluminum production process. Specifically, as the carbon dross content increases from 0.04% to 1%, the electrolyte resistivity rises from 1% to 11%, leading to a continuous rise in the cell temperature. Concurrently, the accumulation of carbon dross leads to a decrease in the vertical current and an increase in the horizontal current within the cell. This altered current distribution causes the horizontal current to pass through the side of the electrolytic cell. In severe cases, the current forms a direct pathway between the cathode and anode, increasing the heat input into the cell and resulting in a “hot cell” phenomenon.^24^ The surface heating of the electrolytes can also cause falsely elevated cell temperature readings, thereby affecting the automated control of the cell temperature. Moreover, carbon dross increases the viscosity of the electrolyte and reduces its fluidity, which leads to a decrease in the solubility, diffusion rate, and other dissolution properties of alumina in the electrolytes, negatively affecting the productivity of the aluminum electrolysis process. The presence of carbon dross compromises the wettability of the carbon anode by the electrolyte, which can induce an anodic effect. This effect further raises the cell temperature and complicates the stabilization of the electrolytic cell temperature. Additionally, the accumulation of carbon dross at the bottom of the carbon anode or its surroundings can lead to the formation of long protrusions on the anode's bottom or side. These protrusions can cause oscillation in the electrolyte voltage, fluctuations in the electrolyte, and splashing of the electrolyte onto the discharge port, where it can cool and form solid deposits. This ultimately affects the accuracy and smoothness of the alumina feeding process.

##### 2.2.1.2 Increase in production costs

The presence of carbon dross has a significant impact on production costs through multiple pathways. Firstly, the increase in the electrolyte resistance due to carbon dross leads to higher cell-voltage levels. This, in turn, converts a portion of the electrical energy into heat rather than productive energy, thereby increasing electrical consumption. Secondly, the elevated cell temperature caused by carbon dross accelerates the oxidation of the carbon anode, which results in increased anode costs. Moreover, several factors related to carbon dross contribute to the shortened service life of the electrolytic cell:^24^ (1) the increases in cell temperature caused by carbon dross lead to sodium absorption in the cathode, causing it to swell and degrade more rapidly. (2) The accumulation of carbon dross can cause side leakage and damage to the furnace chamber of the electrolytic cell. (3) The inhibition of alumina dissolution by carbon dross causes alumina to settle at the bottom of the cell, reducing the lifespan of the cell bottom area. (4) Frequent occurrences of the anode effect disrupt the orderly operation of the electrolytic cell and even lead to temporary shutdowns. The reduced service life of the electrolytic cell increases depreciation costs. Additionally, the removal of carbon dross results in the loss of some electrolytes, thereby increasing the consumption of cryolite and the cost of raw materials. When a large amount of carbon dross is produced, it needs to be salvaged and removed regularly, which increases the labour cost.

#### 2.2.2. Hazards to the ecological environment

**Fig. 4:**
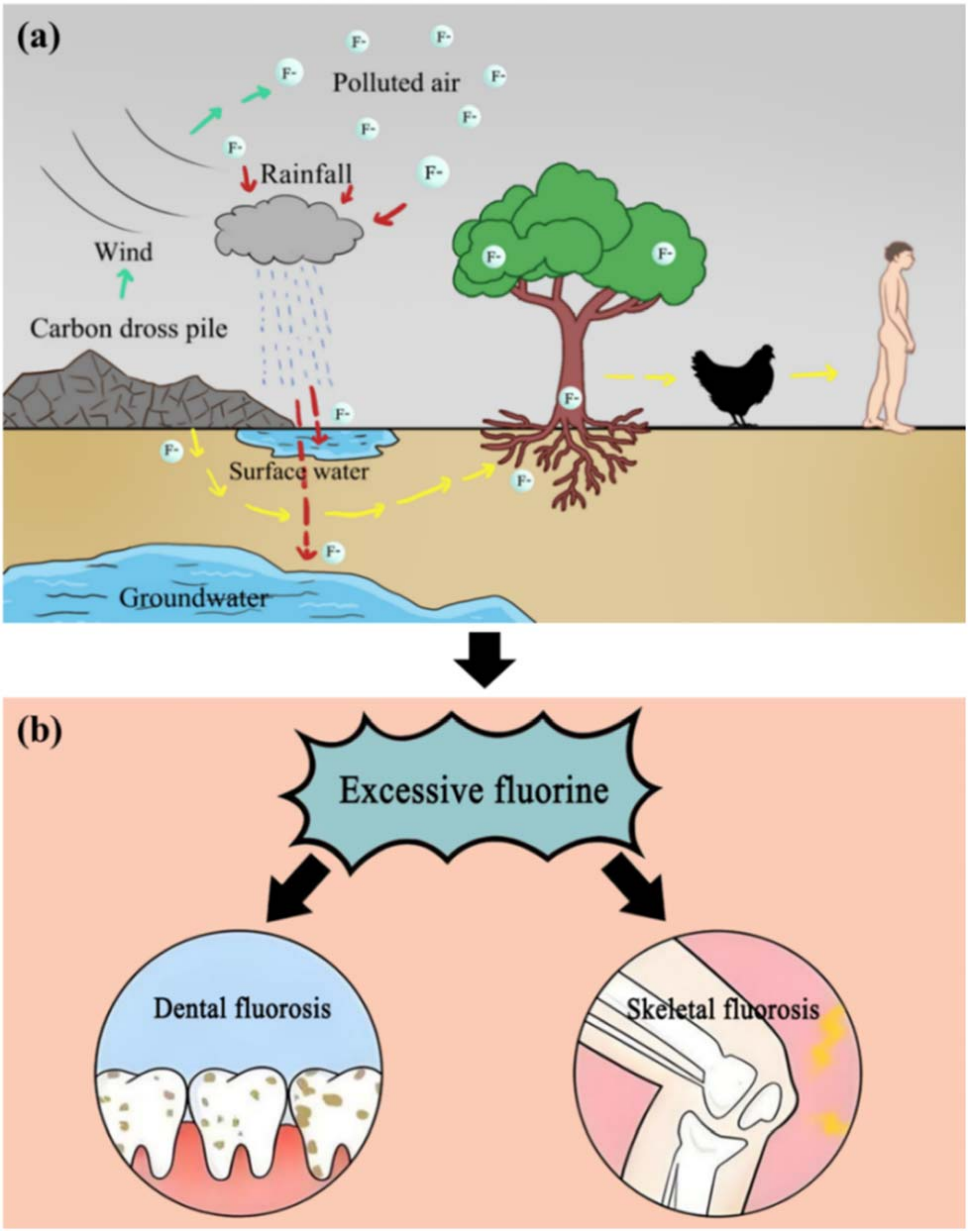
Fluoride hazards to humans, plants and animals. (a) Fluorine causes adverse effects on terrestrial ecosystems; (b) dental fluorosis and skeletal fluorosis.

If carbon dross is not handled appropriately and disposed of properly, its harmful components can infiltrate the environment through various pathways. Carbon dross that is piled up arbitrarily can not only contaminate the atmosphere through wind-blown dust but also pollute water sources. The soluble F^−^ within it can be readily leached by rainwater, seeping into and contaminating both surface water and groundwater. Furthermore, harmful substances in carbon dross are gradually released into the soil, absorbed by plants, and integrated into the food chain, ultimately posing a severe threat to the health of animals and humans (Fig. 4(a)). Specifically, F^−^ primarily harms plants by reducing the efficiency of photosynthesis,^25^ a process that inhibits the synthesis of macromolecules essential for plant growth, weakens enzymatic activity, disrupts metabolic processes, damages plant structures, and, in severe cases, even leads to plant necrosis. On the other hand, when F^−^ enters the animal body, it reduces the survival rate of beneficial bacteria, thereby weakening animal vitality and increasing mortality.^26^ Additionally, fluoride pollution adversely affects soil aggregates; it reacts with soil colloids to form complexes that gradually accumulate in the soil, blocking soil pores, impeding water infiltration, interfering with the activities of soil microbial communities, and ultimately leading to soil degradation.^27,28^ For humans and animals, excessive fluoride intake can damage teeth and bones and even harm multiple body systems, causing systemic diseases.^29^ Among them, the most common diseases caused by fluoride pollution are dental fluorosis and skeletal fluorosis (Fig. 4(b)). The ecological damage caused by carbon dross is therefore widespread, long-term and latent, causing lasting and unrecoverable adverse effects.^30^

### 2.3. Waste of resources

Since carbon dross is classified as hazardous waste, its disposal in landfills poses an ongoing risk of environmental pollution and results in the wasteful discarding of its carbon and electrolyte content. The main components of carbon dross are all valuable materials. The recovery of carbon directly reduces the carbon emitted during aluminium production, reduces human consumption of natural carbon, and helps the world achieve the “carbon peak” and “carbon neutral” goals. The electrolyte components of carbon dross are mainly cryolite, which is key for aluminum electrolysis to occur at a temperature much lower than the melting point of alumina. NaF, AlF_3_, KF, and LiF in the electrolytes are important components of aluminum electrolysis electrolytes. The electrolytes also contain a portion of alumina, which is the raw material for aluminum electrolysis. Thus, if carbon dross can be recycled, the resources and energy consumed in electrolytic aluminium production, the production costs of the enterprise, and the exploitation of natural resources will be reduced. The process of resource utilization also promotes the green and sustainable development of different resources.

## 3. Research progress on pollution–prevention and control technologies for carbon dross

The prevention and control of solid waste pollution must adhere to the principles of clean, cycle and control. It is imperative for all units and individuals to implement strategies aimed at minimizing waste generation, promoting comprehensive recycling, and reducing the harmful impacts of waste.

### 3.1. Research on clean technologies

Clean production is crucial for reducing environmental pollution and conserving natural resources. It helps industries minimize waste generation and promotes sustainable practices for a healthier planet. Throughout the chain of electrolytic aluminium production, conducting clean production processes and reducing the amount of carbon dross generated at the source are prioritized to prevent carbon dross from polluting the environment. The following measures can be taken during aluminium electrolysis.

#### 3.1.1. Producing a high-quality carbon anode

High-quality carbon anodes with low resistivity, high conductivity, and minimal slag generation are produced using high-purity calcined coke aggregates, binders with low impurity content, and efficient dross treatment methods. The raw material formulation, particle-size distribution, and geometric design are subsequently optimized.^20,31^ Finally, vibration forming, roasting parameters, and treatments before and after roasting are precisely controlled to ensure low air permeability, high CO_2_ reactivity, and excellent oxidation resistance, thereby achieving superior and consistent anode performance.

#### 3.1.2. Improvement in controlling the electrolytic process

A properly controlled electrolyte level inhibits cover material melting, anode air oxidation, and slag formation. Regular composition and molecular ratio analyses enable precise fluoride supplementation, ensuring compositional stability and optimal liquidus temperature. Studies have indicated that suitable potassium and lithium concentrations enhance aluminum production, whereas excessive levels impair properties, such as crystallization temperature, conductivity, and alumina solubility, adversely affecting process metrics like cell temperature and current efficiency.^32,33^ These measures improve wettability differentiation between carbon dross and electrolytes, promote self-cleaning behavior, and support efficient separation.

#### 3.1.3. Improvement at the operational level

##### 3.1.3.1 Improve the operating level of the anode exchange

Single anode and double anode exchange methods are available for the replacement of consumed anodes.^34^ Inadequate handling leading to anode exposure accelerates oxidation and increases carbon dross generation. After replacement, it is essential to strictly adhere to standard procedures when reapplying thermal insulation materials. Specific requirements include limiting the cover-opening time to within 20 minutes, transferring spent anodes to cooling boxes within 2 minutes, preheating new anodes to no less than 300 °C, maintaining a distance of 40 ± 5 mm from the anode bottom to the aluminum bath, filling the periphery with three layers of electrolyte totaling at least 120 mm in thickness, covering with an 80–100 mm layer of alumina, and ensuring hood plates are overlapped by at least 20 mm with all seams properly sealed. These measures are implemented to minimize the formation of carbon dross.

##### 3.1.3.2 Improve the operating efficiency of salvaging carbon dross

Optimizing the frequency and volume of dross removal helps maintain a balance between generation and recovery, promoting electrolyte stability and resource efficiency. Introducing mechanized automatic salvage systems can reduce electrolyte entrainment in dross, shorten process time, limit air exposure of the electrolytic system, and lessen worker exposure to harsh conditions. Automation, thus, lowers the labor intensity and reduces dross output.

##### 3.1.3.3 Standardize the daily maintenance operation

Fine-grained covering materials should be used to create a tight seal at the anode and middle seam, applied evenly and compactly to prevent hollow spaces. Special attention should be given to anodes near the material inlet, aluminum outlet, and flue exit. The phenomenon of anode oxidation found at the liquid aluminum outlet and flue outlet, as well as the flaming phenomenon caused by the large surface collapse of covering material on the electrolytic cell, should be dealt with in a timely manner.

### 3.2. Research on cycle technologies

Solid waste that cannot be avoided should be recycled. This approach promotes the recycling of solid waste, which refers to the direct use of solid waste or the use of technology to extract or transform solid waste into useful resources or energy. Cycle technology has the advantages of high environmental efficiency, high production efficiency, low cost and low energy consumption, and it is a sensible response to the “carbon peak” and “carbon neutral” policies.

Carbon dross contains approximately 25–60% carbon and 40–75% electrolytes, with a trace amount of approximately 0.5–1% aluminium. The recovery and recycling of carbon resources from carbon dross can mitigate carbon emissions in the electrolytic aluminium industry. The primary electrolyte constituent in carbon dross is cryolite (Na_3_AlF_6_), which finds extensive applications in mining, metallurgy and ceramic applications. In addition to its use as a solvent for electrolytic aluminium production, it can also be used as a wear-resistant additive for grinding products,^35^ a deoxidiser for casting,^36^ a flux for welding,^37^ a filler for ceramics, and an insecticide for pesticides.^21^ In recent years, researchers have conducted many studies on the comprehensive utilisation of carbon dross, and the main publicly reported treatment technologies include flotation, roasting, vacuum smelting, alkali fusion and leaching.^12^

#### 3.2.1. Flotation method

**Fig. 5:**
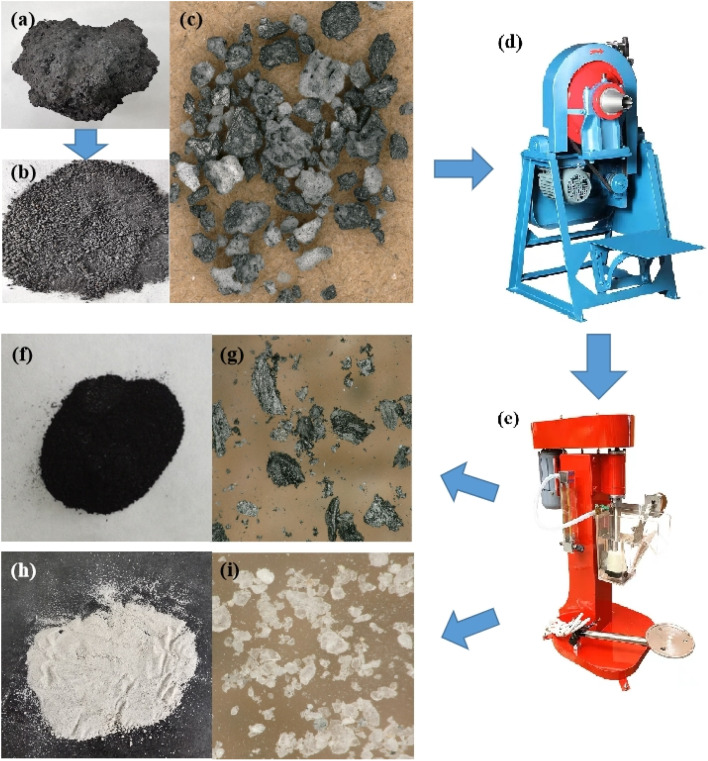
Diagram of the carbon dross flotation experimental process and product. (a) Massive carbon dross salvaged from the aluminium electrolytic cell. (b) Crushed carbon dross. (c) Crushed carbon dross under an ultra-depth three-dimensional microscope (UTMS). (d) Conical ball mill. (e) Single-cell flotation machine. (f) Carbon powder after flotation. (g) Carbon powder after flotation under UTMS. (h) Electrolytes after flotation. (i) Electrolytes after flotation under UTMS.

The flotation method exploits the difference in wettability between electrolytes and carbon in a nonpolar oil system to separate them through aeration.^14^ This process yields carbon powder with a low fluoride content and electrolytes with a low carbon content. The carbon powder can be used in the production of carbon products, such as anode protection rings or additives for anode preparation, while the electrolytes can be returned to the electrolytic cell to replenish losses of cryolite or anode-covering material. The common experimental process of carbon dross flotation research is shown in Fig. 5. Li *et al.*^38^ used kerosene as a collector, terpene oil or terpineol oil as a frother, and water glass as an inhibitor for the flotation of electrolytic aluminum carbon residue from an aluminum smelter in Henan, China. They found that the optimal flotation conditions were 70% of the ground particles with a size of 200 mesh, an impeller speed of 1700 rpm and a slurry concentration of 25%, which yielded carbon powder with a carbon content greater than 85%.

Flotation effectively recovers most resources from carbon dross, enabling the reuse of products in electrolytic aluminum or other industries. It offers advantages such as low equipment requirements, low processing costs, minimal labor needs, and improved efficiency in comprehensive resource utilization, making it promising for industrialization. However, variations in the characteristics of carbon dross from different electrolytic aluminum plants and differences in flotation processes often result in low separation efficiencies and high impurity levels in the flotation products. Specifically, if the carbon content in the electrolyte product exceeds 5%, it can only partially replace cryolite in aluminum electrolysis. Moreover, if the electrolyte inclusions in the carbon powder are significant, the product becomes hazardous waste with leaching toxicity (F^−^ concentration in the leachate > 100 mg L^−1^), as defined by the “Hazardous Waste Identification Standard-Leaching Toxicity Identification” (GB 5085.3–2007). Additionally, flotation-efficiency fluctuations arise from (1) variations in the feed ore properties or slurry characteristics altered by equipment wear (*e.g.*, ball mill steel balls); (2) accumulation of impurities (oils, suspended solids, soluble salts, *etc.*) in process circulating water, impairing reagent efficacy; and (3) suboptimal control of key parameters, including aeration rate, bubble size, flotation time, agitation speed, slurry density, pH, and temperature.

**Fig. 6:**
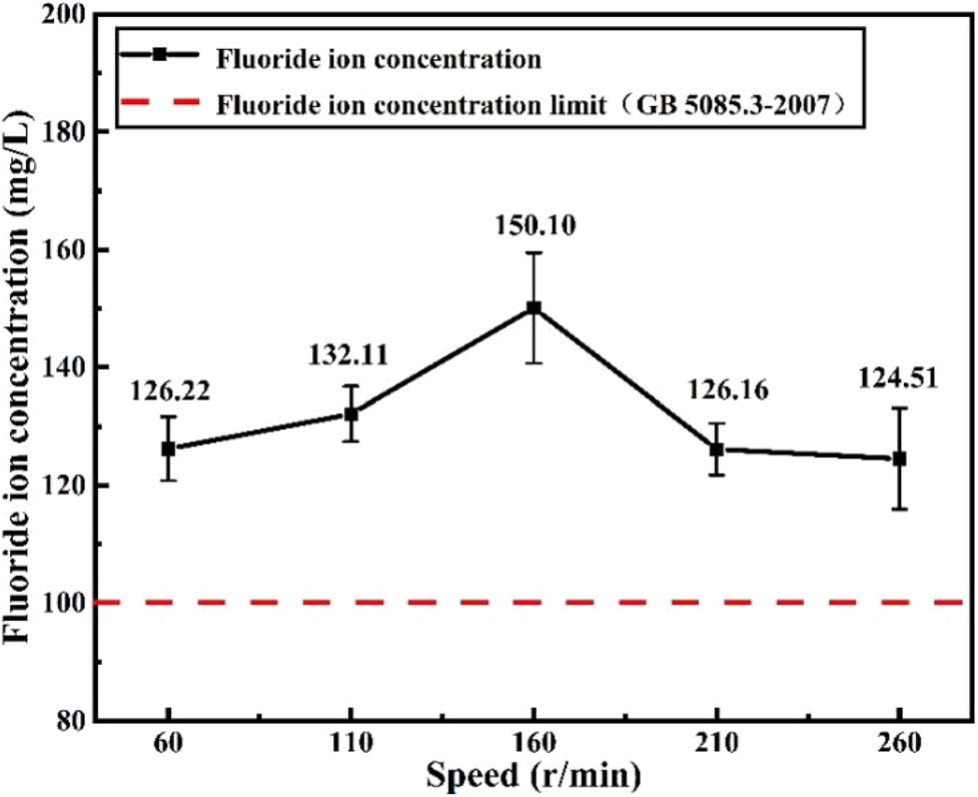
Fluoride-ion concentrations in the leaching solutions of carbon powder collected from a carbon dross flotation industrial line (note: the shaking speeds specified in “HJ 557-2010” is 110 ± 10 rpm).

Flotation stood as the sole industrially utilized technology for carbon dross recycling in China before 2016, attributed to its benefits, including physical separation at ambient temperature and pressure, a well-established technology base, and cost-effectiveness. In China, the largest primary aluminum production country, several carbon dross flotation production lines were built in some electrolytic aluminum plants to treat carbon dross. We conducted leaching toxicity tests on carbon powder products from a carbon dross production flotation line based on the “Leaching Toxicity Leaching Method for Solid Waste Horizontal Oscillation Method” (HJ 557–2010). However, according to the results in Fig. 6, the carbon powder products obtained from the industrial flotation production lines are still hazardous waste, and technical upgrading is needed to reduce the levels of fluorine impurities in the carbon powder. However, the existing flotation research on carbon dross is mostly focused on the optimization of flotation process conditions, without in-depth flotation theory research, and it fails to provide technical guidance for production at the mechanism level.

#### 3.2.2. Roasting method

The roasting method involves heating carbon dross to burn off the carbon content while minimizing electrolyte decomposition or volatilization, resulting in purer electrolytes. Chen *et al.*^39^ studied optimal combustion aid, dispersant dosages, roasting temperature range, and rotary kiln speed. This method can achieve electrolytes with over 99% purity, which can be reused in electrolytic cells. Kang *et al.*^40^ conducted low-temperature cyclic roasting experiments, achieving a 97.6% carbon removal rate at 750 °C after secondary roasting. Zhou *et al.*^41^ used fluidized roasting with a bubble fluidized bed, finding the ideal temperature range for roasting to be 565–725 °C. While roasting produces pure electrolytes, it has several drawbacks. It requires precise temperature control. The excessive heat can cause the electrolyte to melt and bond to the inner walls of the roasting equipment or lead to the volatilization of the electrolyte. Too low a temperature can lead to incomplete combustion, reducing purity and efficiency. Additionally, the method requires a sufficient supply of oxygen, combustion aids, dispersants, and catalysts, leading to high energy consumption and raw material costs.^42^ The combustion process also fails to recover carbon resources, increasing CO_2_ emissions. Moreover, high temperatures and uncontrolled reactions can produce corrosive fluorine fumes, damaging equipment and raising manufacturing and maintenance costs.

#### 3.2.3. Vacuum smelting method

The vacuum smelting method involves treating carbon dross at high temperatures within a vacuum furnace, leveraging the difference in volatilization temperatures between electrolytes and carbon. During this process, the carbon dross is placed at the high-temperature end of the vacuum furnace. The electrolytes vaporize at high temperatures and low vacuum, rise towards the top of the furnace, and then condense at the cooler end. Meanwhile, the carbon remains at the high-temperature end, facilitating the separation of the two components.^7^ Chai *et al.*^43^ conducted an exploratory study of the separation of carbon dross by vacuum smelting and mainly investigated the effects of different experimental conditions on electrolyte separation. The results indicated that the best process conditions required a vacuum of 5 Pa, a reaction temperature of 950 °C, raw material particle sizes of 0.5–1 mm, and a reaction time of 4 h. The separation rate for fluorides was above 83%, and the carbon content in the bottom residue was above 74% under the optimal conditions. The separation of carbon dross *via* vacuum smelting is currently in the experimental research phase, with numerous practical challenges in engineering applications still awaiting resolution.

#### 3.2.4. Alkali fusion method

The alkali fusion method, primarily aimed at recycling carbon powder from carbon dross, involves reacting molten alkali with the electrolytes in carbon dross at high temperatures. This process removes impurities, such as fluoride, oxides and aluminosilicates, yielding purer carbon powder after washing, neutralisation and drying.^44^ Yang *et al.*^45^ processed carbon dross by the alkali fusion method, determining the optimal reaction conditions through a single-factor test and an orthogonal test: the reaction temperature was 600 °C, and the reaction time was 6 h. Carbon powder with a 99.10% carbon content was obtained and reused as a lithium battery anode. Zhao *et al.*^46^ used a high-temperature alkali melting process, and Tian *et al.*^47^ used a high-temperature activation-chemical leaching-mechanical ball milling process for treating carbon dross. The recycled products were used to prepare lithium battery anodes or cathodes. The alkali fusion method is a breakthrough attempt to treat carbon dross, and this is a new technology that can provide high-purity carbon products. However, its drawbacks include high alkali consumption, extended reaction times, elevated reaction temperatures, and substantial treatment costs. Furthermore, recycling the electrolytes post-alkali fusion necessitates further investigation.

#### 3.2.5. Leaching method

The leaching method uses reagents, such as acids, alkalis, aluminum salts, or saline solutions, to react with carbon dross, causing elements like fluorine, aluminum, and sodium in the carbon dross to undergo chemical reactions and enter the gas or liquid phases. Then, the carbon in the leaching residue is purified.^48^ Kondrat'ev *et al.*^49^ enhanced the F-leaching rate by treating carbon dross under high-pressure alkaline leaching conditions. Additionally, this method can convert insoluble impurities, such as SiO_2_ and Al_2_O_3_, into soluble sodium metasilicate and sodium aluminate, thereby increasing the carbon content of the leaching residue. Yang *et al.*^48^ treated the carbon dross under the optimal conditions of 4 mol L^−1^ hydrochloric acid concentration, a liquid-to-solid ratio of 25 mL g^−1^, leaching temperature of 140 °C, and leaching time of 270 minutes, achieving a carbon purity of 97.3% in the recovered product. The carbon dross leaching method has advantages, such as a high separation efficiency and a high recovery rate. Nevertheless, the leaching method faces several challenges. Further treatment of the leachate is required to recover valuable components, and the complex treatment of acid-alkaline waste liquids and the emission of harmful gases limit its widespread application.^14^ Additionally, the purity and added value of the recovered products during the leaching process need to be improved. Future research needs to seek a balance between improving the leaching efficiency, reducing environmental risks, and enhancing the resource recovery value to promote the sustainable development of carbon dross leaching technology.

#### 3.2.6. Emerging and hybrid processing technologies

In recent years, a variety of forward-looking processes have been proposed and have demonstrated significant progress. The “chemical leaching–high-temperature graphitization” process employs alkali leaching (15% NaOH, L/S = 10 : 1), followed by acid leaching (15% HCl, L/S = 15 : 1) to remove the vast majority of fluorides and oxides from anode carbon residue. Subsequent graphitization treatment at 2800 °C yields the anode material with a carbon purity of up to 99.9%, exhibiting a first discharge specific capacity of 359.1 mA h g^−1^ and excellent cycling performance.^46^ The aluminum–fluorine complex closed-loop process utilizes two-stage leaching (aluminum salt leaching: 50 °C, L/S = 14, 24 h; alkali leaching: 100 °C, NaOH = 100 g L^−1^), achieving leaching rates exceeding 93% for fluorine, sodium, and aluminum. The final products included carbon residues with a purity of 85.84% and a mixture of AlF_3_ and Al_2_O_3_ with a purity of 95.94%, and the process featured wastewater recycling and zero liquid discharge.^50^ In the mechanically activated assisted flotation method, mechanical activation (300 rpm, 90 min) combined with flotation (using sodium silicate, kerosene, and terpineol as reagents) produces a concentrate with a carbon grade of 90.58% and a recovery rate of 77.87%, along with a fluoride removal rate of 96.26%.^51^ Furthermore, the low-temperature fluorination roasting-water leaching technique (using NH_4_F as a fluorinating agent, 20% dosage, 70–300 °C) can treat spent carbon anode with a high carbon content. This process enhances graphite purity to 99.98%, allows for the recovery of valuable metals, and yields regenerated graphite anode material with performance comparable to those of commercial products (capacity: 340.9 mA h g^−1^, ICE = 92.13%).^52^ Future research should place greater emphasis on process intensification and system integration.

In addition, this study compared the key performance indicators of various carbon dross treatment technologies (Table S1 in SI), thereby providing a scientific reference for technological selection in this field.

### 3.3. Research on control technologies

Control technology for solid waste encompasses engineering solutions that utilize physical, chemical, or biological treatments to reduce or eliminate harm to human health and the natural environment. Most electrolytic aluminium plants in China have collaborated with enterprises specializing in hazardous waste treatment to prepare secure landfills for the control and disposal of carbon dross. Some countries, such as Norway and Spain, have also chosen secure landfill sites as the final destinations of carbon dross. Disposal through secure landfilling seems simple, and the standardized design and use of secure landfills can completely prevent carbon dross from harming the ecosystem. Nevertheless, this method has its drawbacks, including land occupation, resource wastage, high disposal costs, and significant long-term maintenance expenses.^53^

Other harmless treatment methods for carbon dross include wet and thermal defluoridation processes, both of which involve crushing and grinding the dross into powder.^38^ Wet defluoridation involves mixing carbon dross powder with a strong calcium oxide solution to react fully, forming a calcium fluoride precipitate for fluoride removal.^54^ Thermal defluoridation refers to the removal of fluoride at high temperatures.^55,56^ The carbon dross is mixed with a certain proportion of limestone and then roasted in a rotary kiln. The carbon is burned in this process, and most of the fluoride is converted into stable solid components, which are mainly calcium fluoride and have low water solubility.^57^ The thermal process offers larger-scale treatment compared to the wet process, with by-products usable in the construction of materials for sustainable resource utilization. However, its high cost restricts its widespread application.

## 4. Application progress of pollution prevention and control technologies of carbon dross

### 4.1. Progress in the application of clean technologies

Reducing the amount of carbon dross in the production process is a way to decrease its generation at the source. Clean technologies, including improving the production process, optimising the physical and chemical properties, and enhancing the technical conditions for electrolytic production, are the main ways for reducing carbon dross generation.

#### 4.1.1. Optimisation of the anode production process

Improving the antioxidant performance of the anode helps reduce the carbon dross generated during aluminum production. Chen *et al.*^58^ discovered that by adding a compound antioxidant additive to a prebaked anode, the anode air reaction and CO_2_ reaction residual rates were greatly improved, resulting in a reduction in the generation of carbon dross. Further optimisation of the anode production process can improve the anode quality and reduce carbon dross generation. Zhang^31^ studied the optimisation of the carbon anode structure by increasing its height, refining the top shape, and reducing the depth of its carbon bowl in an aluminium electrolysis enterprise. These measures lead to reduced carbon-anode consumption and lower carbon dross production.

#### 4.1.2. Improving the process conditions of electrolytic aluminium production

##### 4.1.2.1 Regular analysis and optimisation of the electrolyte composition

The composition of electrolytes has a direct impact on their viscosity, influencing their fluidity and separation efficiency from carbon dross. An optimal quantity of K-cryolite demonstrates a significant capacity for alumina solubility. Notably, LiF stands out as the sole fluoride compound that enhances electrical conductivity by dissociating into small Li^+^ cations, thereby augmenting the electrical conductivity of cryolite melts.^59,60^ In some electrolytic aluminum plants in northern China, it is observed that the alumina-introduced lithium and potassium contents in electrolytes accumulate to approximately 10% and 5%, respectively, posing a substantial threat to the stability of the production process. Consequently, regular analytical assessments are imperative to maintain the compositional stability of electrolytes throughout production, with timely replenishment of AlF_3_ or removal of excess interfering elements based on the analytical outcomes.

##### 4.1.2.2 Use of additives

Additives can be incorporated into the electrolytic cell to enhance the wettability disparity between the electrolytes and carbon dross, simultaneously augmenting the interphase tension between them. This facilitates the floating of carbon dross on the electrolyte surface, thereby accelerating the separation process between the carbon dross and electrolytes.^61^ Presently, AlF_3_, MgF_2_, and CaF_2_ are commonly employed as additives. These additives reduce the initial crystallisation temperature and impede the penetration of the electrolytes into the carbon material.^62^

##### 4.1.2.3 Keep a reasonably low cell temperature

When the cell temperature is reduced to a relatively reasonable low temperature, the surface tension of the electrolytes increases, the separation of the carbon dross can be promoted, and the accumulation of carbon dross can be reduced.^63^ In production processes, various factors affect the cell temperature, such as the Al_2_O_3_ feed rate, cell voltage, electrolyte composition, and anode effect. To maintain a reasonably low cell temperature, it is crucial to continuously monitor the production conditions and technical indicators of the cell, and to adjust the electrolytic environment according to the monitoring data. However, it is worth noting that low cell temperatures should be avoided in practical industrial applications, as they can impair the electrolyte's mobility and cause it to become viscous, potentially severely disrupting the production process. Consequently, it is essential to maintain the cell temperature within an optimal range of 925 °C to 945 °C, which represents a low but functional level that ensures both good operational efficiency and electrolyte stability.

##### 4.1.2.4 Keep a reasonable electrolyte height

In production, if the molten electrolyte height is too low, its alumina solubility diminishes, leading to excessive alumina precipitation at the bottom of the cell. This disrupts the normal operating state of the electrolytic cell and shortens its lifespan. Conversely, a high electrolyte height tends to create “cavities” between the carbon anode and the upper insulation material, facilitating the flow of the electrolytes. The washing effect of electrolytes accelerates the surface oxidation and the falling of debris off the carbon anode, resulting in an increase in the carbon dross quantity. Based on production experience, it has been determined that the electrolyte height should be maintained between 18 to 20 cm, depending on factors such as the depth of the cell, the anode height, and the actual production yield in the electrolytic plant.

#### 4.1.3. Improvement at the level of production operations

##### 4.1.3.1 Improve the operational efficiency of anode exchange

During production, it is essential to adhere to the principle of keeping anodes free from old shells and caking, by using appropriate insulation materials. Enhanced inspections of insulation materials are necessary to guarantee normal anode operation. If the insulation material is too thin, the upper surface of the anode is prone to oxidation due to air leakage and slag drop.^64^ If the insulation material is too thick, the cell temperature rises due to reduced heat loss. The thickness of the insulation material on the anode should be kept in the range of 150–180 mm. The insulation material should be evenly covered, remaining flat and fully exposed, except for the middle seam and the side of the anode, which contributes to the stable operation of electrolytic aluminum production.

##### 4.1.3.2 Circulation of carbon dross in electrolytic cells

In actual production, when the anode and cathode perform well and only a small amount of carbon dross is produced, it can be left in the cell without treatment or an appropriate amount of carbon dross can be added. When the anode and cathode are used for a long time and their physical and chemical properties degrade, the amount of carbon dross generated increases and cannot be consumed due to limited mass transfer. In this case, the excess carbon dross must be periodically removed, and the anode paste or anode should be adjusted or replaced as needed. Furthermore, an appropriate amount of carbon dross in the electrolytic cell can replenish the fluoride salts in the electrolyte and help regulate the alumina feeding. A moderate amount of carbon dross can also reduce the cell temperature and stabilize both the electrolytic environment and parameters. Therefore, in practice, the temperature of the electrolytic cell can be controlled by adding an appropriate amount of carbon dross when necessary.

##### 4.1.3.3 Application of graphitized cathodes

Large-sized graphitized cathode carbon blocks have been produced in China by controlling furnace conditions, such as roasting temperature, material loading, discharging operations, and roasting process quality. GO–TiB_2_-modified bitumen can enhance the carbon-bonding phase of graphitized cathode bitumen, improving its mechanical stability during electrolytic expansion and creep, and enhancing its resistance to electrolytic expansion/creep and elasticity.^65^ Graphitized cathodes exhibit excellent durability under the erosion of various elements, including sodium and vanadium, and their improved permeability resistance reduces the formation of carbon dross caused by the expansion and rupture of cathode carbon blocks. Graphitized cathodes can extend the life of electrolytic cells from about 1500 days to more than 3000 days,^47^ while also reducing the amount of carbon dross produced by cathode slag during use.

##### 4.1.3.4 Research and application of automatic salvage devices

Automatic carbon dross salvage devices can greatly enhance salvage efficiency and reduce the labour intensity of workers. CHINALCO, as well as some private electrolytic aluminium enterprises, has conducted research on the development and improvement of automatic carbon dross–salvage devices.^22^ However, there have been no reports on the large-scale promotion and application of these devices yet.

### 4.2. Recent status of application of cycle technology

The primary goal of electrolytic aluminium enterprises is to recover electrolytes from carbon dross in order to achieve its resource utilization. The primary technology used is flotation. Some enterprises plan to adopt the roasting method to treat carbon dross, but no successful cases have been reported yet. The vacuum smelting method and alkali melting technology, due to their inherent technical flaws, are seldom utilized in practical industrial applications.

#### 4.2.1. Application status of the flotation method for treating carbon dross

**Fig. 7:**
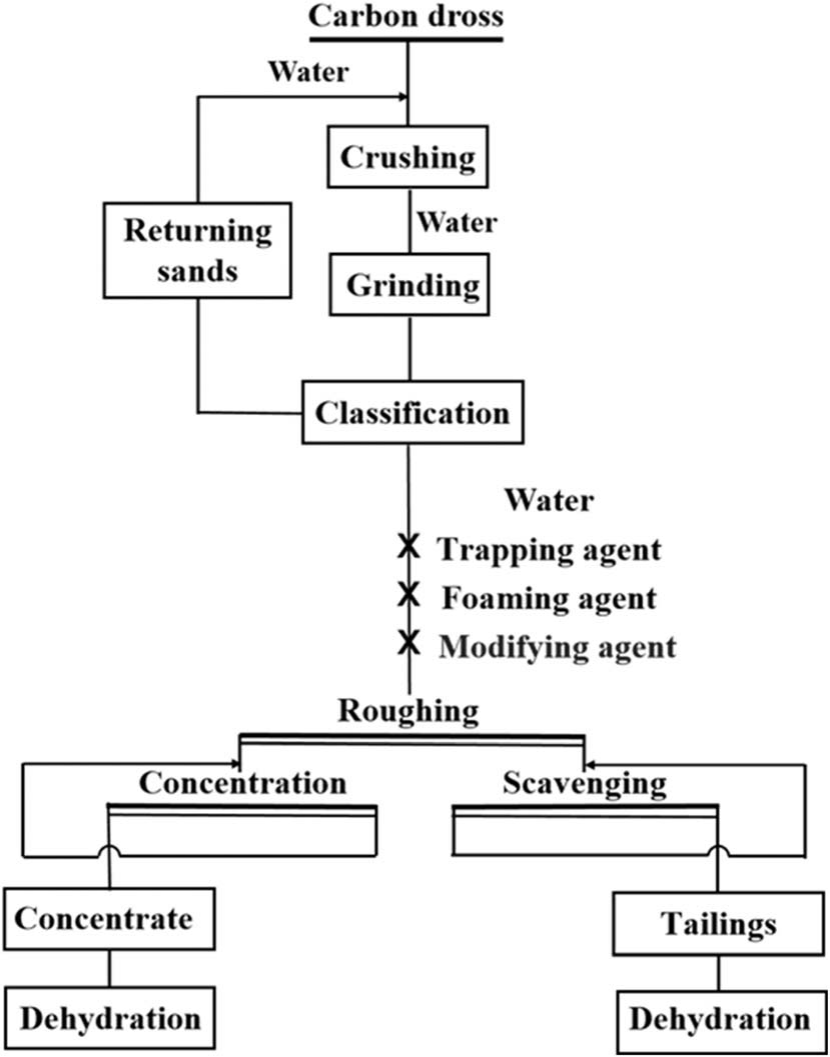
Flotation process for carbon dross.

The carbon dross flotation process, a physical separation technique, comprises several key steps: crushing, grinding, classification, flotation, and dewatering, as shown in Fig. 7. Initially, carbon dross is extracted from the electrolytic cell and cooled. Subsequently, coarse separation removes large aluminum fragments and debris, followed by crushing and grinding to achieve optimal particle sizes. This prepares the carbon and electrolytes for monomer liberation. After grinding the slurry to the required concentration, flotation chemicals are introduced, and the sample is agitated in the flotation tank for foam flotation. Carbon particles rise with the foam and are scraped off, while the electrolytes settle at the bottom, completing the flotation separation. Concentrate (carbon powder) and tailings (electrolytes) undergo dewatering and drying for reuse. Kang^66^ reported that the Qingtongxia Aluminium Group Company used flotation technology, including grinding and classification, flotation, and dewatering, for the recovery of electrolytes and alumina from carbon dross. However, industrial processes have disadvantages, such as high impurity content, a complex process flow, and the need for secondary wastewater treatment. Wei^67^ reported that Shanxi Zhaofeng Aluminium Power Co., Ltd in China used a combined process of flotation decarbonization, bleaching fluorine preservation and strong magnetic iron removal to recover recycled cryolite from carbon dross, and the excess carbon powder was used for preparing anode protection rings to reduce the erosion of the electrolytes by the anode steel claws.

**Fig. 8:**
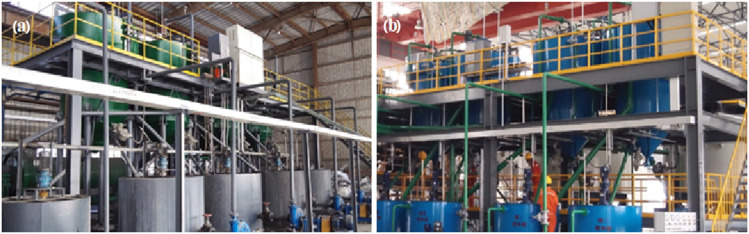
Carbon dross flotation production line. (a) A 6 kt a^−1^ treatment line in Henan, China and (b) a 20 kt a^−1^ treatment line in Liaoning, China.

Huo *et al.*^12^ combined selective grinding technology with driveless flotation technology and replaced the traditional inefficient flotation process with the efficient separation of carbon and electrolytes in carbon dross. This improved technology greatly reduced the impurity content in the flotation product. The technology was successfully transformed in 2012 at Chiping Xinyuan Aluminium Co., Ltd in Shandong Province. A 10 kt a^−1^ electrolyte carbon dross flotation line was successfully constructed at Rare Earth Aluminum Co., Ltd, Baotou, east HOPE. In 2016, Huo *et al.*^12^ developed a high-efficiency collector agent based on the original technology to further improve the separation of electrolytes and carbon, and their achievements were successfully reproduced by Henan Zhongfu Aluminium Co., Ltd and Liaoning Yingkou Zhongwang Aluminium Co., Ltd (Fig. 8). The construction cost of the treatment line is about RMB 5000 per ton of carbon dross treatment capacity. The production cost is RMB 400–600 per ton of carbon dross, while the product (including the recovered electrolytes and carbon powder) costs about RMB 1500–2000. Combined with the above, the construction cost could be recouped within 5 years through rough estimation. Furthermore, the payback period would be reduced to about 3 years if the saved secure landfill disposal cost (cost in China in 2025: RMB 700 per ton carbon dross) is also calculated within the profit range. The investment in a 6 kt a^−1^ carbon dross treatment line in Henan under the EPC + O mode was recouped within 30 months. Thus, the improved flotation technology solves the issues of carbon dross treatment and disposal and provides good economic, social and environmental benefits.

#### 4.2.2. Application status of the roasting method for carbon dross treatment

Theoretical and experimental studies have shown that the electrolyte product obtained by roasting has a high purity and a low carbon content. Some enterprises with high purity requirements for reused electrolytes have tried to conduct semi-industrial applications of the roasting method.

Yunnan Yunlv Chungxin Aluminium Co., Ltd roasted carbon dross at 1000–1300 °C to completely burn the carbon component and obtain molten electrolytes. This technology was identified as a leading technology in China in 2013, but no subsequent applications have been reported. From an environmental impact assessment study published on the Longxi County People's Government website and a news report, it is known that Jiujiang Steel Group Gansu Dongxing Aluminium Co., Ltd does not use the original plan (combined flotation and magnetic separation method) to recover electrolytes from carbon dross but instead uses equipment, such as an electrolyte remelting furnace, to recover electrolytes from carbon dross by heating and remelting.

### 4.3. Recent status of application of control technology

It is very convenient to deliver carbon dross to qualified enterprises for secure landfilling, which has become the choice of many electrolytic aluminum enterprises. With the shortage of land resources and the implementation of new laws and regulations, such as the National Catalogue of Hazardous Wastes, China (From the 2016 Edition to the 2021 Edition and now to the 2025 Edition), the costs of secure landfill disposal increase year by year. This greatly increases the production cost of enterprises, forcing them to step up technical innovation. As for other methods, such as fluoride fixation or fluoride removal technologies, there have been no reports on their industrial application.

## 5. Future trends

As hazardous waste, carbon dross must not be stored or disposed of in open areas. It should be treated on-site within aluminum electrolysis plants or handled by certified hazardous waste management providers. At present, few electrolytic aluminum enterprises in China recycle carbon dross; most store it in newly built on-site warehouses or outsource it to licensed processors while awaiting mature treatment technologies. Nevertheless, controlling carbon dross pollution is a crucial strategic challenge that must be addressed to support the industry's green and low-carbon transformation. Achieving this requires collaborative efforts from government, industry, and society—emphasizing clean production and resource recycling—to drive innovation in both management and technology.

### 5.1. Management system guarantee

The government should establish and improve laws, regulations, emission standards, and management systems while strengthening publicity, supervision, and penalties to systematically promote source reduction and resource recycling. For example, on October 21, 2021, China's National Development and Reform Commission and other agencies released the “Several Opinions on Strict Energy Efficiency Constraints to Promote Energy Saving and Carbon Reduction in Key Areas,” along with the “Action Plan for Energy Saving and Carbon Reduction in Key Industries of Metallurgy and Building Materials (2021–2025)” to enhance energy efficiency and low-carbon development. Additionally, on October 25, 2022, the Zhejiang provincial government launched a three-year action plan aimed at achieving “Zeroing Landfill” of hazardous waste. On the other hand, the government should issue relevant guidance for training relevant scientific researchers, simultaneously provide corresponding subsidies and rewards to enterprises that actively engage in the relevant work,^68^ thereby fostering the rapid development of solid-waste treatment and disposal technologies. Additionally, enterprises should upgrade their equipment promptly, optimize process flows, strictly control the qualities of raw materials,^19^ and effectively implement “reducing from the source” and resource recycling during production.^69^ The society should actively adopt a supervisory role and voice public opinions, becoming the “invisible hand” that promotes green and efficient development within the electrolytic aluminium industry.

### 5.2. Technology development trend

The most effective strategy for eliminating carbon dross generation during aluminum electrolysis is to address the issue at its source, such as through the development of inert anodes. This approach fundamentally prevents carbon by-product formation by replacing traditional carbon-based anodes with non-consumable ceramic materials, thereby achieving zero-carbon-emission aluminum production. In 2018, the Canadian government, Alcoa, Rio Tinto, and Apple jointly established the Elysis Research and Development (R&D) Center to advance industrial research on inert metallic ceramic anodes.^70^ By 2019, the center had produced the first batch of carbon-free aluminum with a purity exceeding 99.7%, meeting all industry standards.^70^ Subsequently, in 2021, industrial inert anodes were installed in a 450 kA electrolysis cell at the Alma Aluminum Smelter in Canada to evaluate the technology's operational efficiency and scalability under full-scale industrial conditions.^70^ According to data from the IAI, taking the global primary aluminium production of 68.3 million tonnes in 2022 as an example, the adoption of inert anode technology in place of traditional processes would reduce carbon dioxide emissions by 11.92 million tonnes, conserve 27.32 million tonnes of high-quality carbon resources, and generate 59.39 million tonnes of oxygen.^71^ The inert anode aluminium electrolysis is a disruptive and foundational technology that has the potential to transform the traditional aluminium electrolysis industry and play a crucial role in achieving carbon neutrality.^72^

However, commercialization faces challenges due to corrosion resistance, mechanical stability, and system integration. While NiFe_2_O_4_-based anodes with metal additives (*e.g.*, Cu, Ni) show promise under lab conditions, corrosion accelerates markedly at industrial current densities (>1.0 A cm^−2^).^73^ Cu–Ni anodes offer high conductivity but suffer from unstable passive films above 900 °C and sensitivity to Al_2_O_3_ concentration fluctuations.^74^ Mechanical limitations include low fracture toughness (∼4.96 MPa m^1/2^) and flexural strength (75–152 MPa) of NiFe_2_O_4_ ceramics, exacerbated by metal-phase agglomeration during sintering.^74,75^ Reinforcement strategies, including using metal meshes, improve conductivity (up to 1546 S cm^−1^) but are vulnerable to corrosion-induced spalling.^75^ Current research focuses on material and process improvements: novel composites (*e.g.*, NiFe_2_O_4_–10NiO–Cu/Ni) with sintering aids (MnO_2_, TiO_2_) achieve near-full density (98.11%) and high conductivity (39.61 S cm^−1^).^74,76^ Process controls, including temperature (850–920 °C), high Al_2_O_3_ content (5.2%), and optimized current density (1.25 A cm^−2^), promote stable surface layers.^73^ However, industrial-scale applications must address scale-up challenges. Simulations of 5 kA electrolytic cells show that a six-anode configuration yields more favorable distributions of the multiphysics fields (electric, magnetic, thermal, and flow) than an eight-anode array, yet thermal-stress concentration still tends to cause anode cracking.^77^ Critical breakthroughs remain necessary in three areas: advanced additives for improved robustness, dynamic corrosion modeling that accounts for inter-electrode distance (<3 cm) and gas films, and alternative electrolytes such as low-melting-point salts. Synergistic advances in both materials and electrolysis cell design are essential to fully realize the industrial potential of inert anodes.

**Fig. 9:**
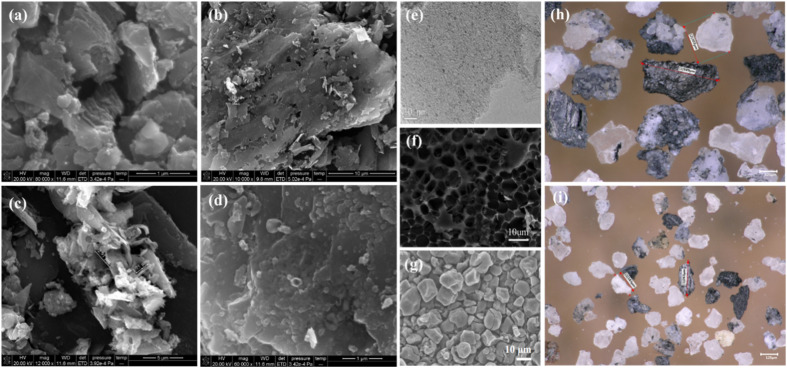
Morphology and applications of carbon dross. (a)–(d) SEM images of carbon dross from different aluminum electrolytic plants. (e) TEM photograph of carbon catalytic supports;^82^ (f) SEM photograph of an efficient carbon-based adsorption material.^85^ (g) SEM image of the deoxygenated porous carbon for capacitors.^86^ (h) and (i) Ultra-depth three-dimensional microscopy image of mechanically liberated carbon dross.

At present, the majority of primary aluminum smelters inevitably generate carbon dross as a by-product during production. The comprehensive recovery of valuable resources from this unavoidable by-product has become an irreversible global trend. Historically, technologies have predominantly concentrated on the recycling of electrolytes due to their significant worth. In the context of global carbon reduction efforts,^78^ the recycling of carbon has become a hot spot for technological research. By observing the carbon dross samples from different aluminum electrolysis plants using SEM (Fig. 9(a)–(d)), it can be clearly seen that due to electrolytic oxidation, secondary reactions and gas generation during the electrolyte erosion process, the morphology of carbon dross becomes loose and porous, featuring certain multidimensional structures. Furthermore, through physical activation,^79^ chemical activation,^80^ or combined physical–chemical activation,^81^ these pore structures can be further opened and expanded. This process increases the specific surface area of the carbon material. It can be used to prepare excellent catalytic carriers (Fig. 9(e)),^82,83^ efficient adsorbent materials (Fig. 9(f)),^84,85^ and electrodes and hyperpolar capacitors (Fig. 9(g)),^86,87^ among other applications. In addition, the recovered electrolytes from carbon dross not only exhibit high reusability in production but also allow for the adjustment of their elemental composition. This includes the removal of impurities such as iron and silicon, as well as the modulation of beneficial elements, like lithium and potassium.^88^ In comparison to the direct adjustment of the electrolyte composition, modifying the recovered electrolytes minimizes the consumptions associated with extraction from the electrolytic cell, cooling, composition adjustment, and remelting processes.^89^ This approach conserves considerable energy and enables continuous adjustment during routine production, thereby maintaining the stability of the electrolyte composition without affecting the smooth operation of the electrolytic production process. It should be noted that the current research on resource recovery processes still lacks investigation into carbon dross liberation. Since carbon dross is extracted from molten electrolytes, it inherently contains complex structures where electrolytes encapsulate carbon particles and infiltrate into pores of varying sizes within the carbon matrix. Consequently, regardless of the employed methods, such as flotation, roasting, vacuum smelting, alkali fusion, or leaching, it is imperative to first reduce the dross from bulk into fine powders. This comminution process facilitates preliminary liberation between electrolytes and carbon components (Fig. 9(h) and (i)), thereby enhancing subsequent treatment efficacy. However, existing studies on carbon dross liberation mechanisms and optimization strategies remain underexplored, with no literature reported to date addressing this critical pretreatment step.

### 5.3. Challenges and prospects for scale-up

Although vacuum melting can cleanly recover metal and electrolytes from carbon slag in the laboratory, its industrial scale-up is challenging. Commercial furnaces must seal and survive >1500 °C under high vacuum, demanding costly, creep- and corrosion-resistant alloys; enlarging the chamber steeply raises heating energy and makes heat transfer the limiting step.^90,91^ Controlling temperature uniformity and vacuum across a massive melt is far harder than the case at the bench scale, while continuous feed of solids and discharge of melts introduce intricate hardware and constant risk of air in-leakage. High electrical power and capital/operating expenses tie economic viability to product value and scale, and no large-scale industrial deployment has been reported. Alkali roasting (or alkali fusion) typically involves mixing the slag with NaOH/Na_2_CO_3_ and reacting at high temperatures; scaling it hinges on reactor design for a fiercely corrosive medium and on downstream wet processing. Molten alkali attacks most metals; thus, costly specialty refractories or nickel-based alloys are mandatory, raising both expense and contamination risk. In industrial rotary kilns or fluidized beds, uniform contact between carbon slag and alkali salt is hard to sustain, leaving zones of incomplete reaction.

To meet these scale-up challenges, future research should prioritize pilot trials and process-integration optimization. Developing novel corrosion-resistant materials, designing high-efficiency reactor configurations, and exploring low-temperature or atmospheric-pressure alternatives are essential steps to move aluminum electrolysis carbon slag treatment from the bench to plant scale.

### 5.4. Practical application verification

The new technologies require industrial experiments at various scales and continuous improvement to better meet the needs of production practice. The successful large-scale industrial application of new technologies depends on both their technical and economic feasibility. Technologies must be evaluated based on whether the technical conditions are controllable and operable, whether they can result in difficult-to-manage secondary pollution, and whether the treatment efficiency and product purity meet the required standards. Among the existing electrolytic aluminium carbon dross–treatment technologies, the flotation method offers significant application potential for the multiple selection and combination of flotation reagents. As a physical separation technique, flotation is conducted entirely in the aqueous phase, without high-temperature or fluorine-containing fume emissions, making it the most ideal method with the greatest research potential at present. From an enterprise perspective, economic benefits are critical, especially if the technology meets industry standards. While flotation has a cost advantage for treating carbon dross, overcoming the technical challenge of achieving higher product purity at the enterprise scale would enhance its economic viability. However, with the increase in the outsourcing disposal cost of carbon dross, other treatment methods are being explored, showing great application potential. Future research directions on carbon dross treatment should move beyond relying on a single technology and explore the integration of multiple methods, organic combinations, and synergistic treatments to maximize their strengths and minimize weaknesses. Moreover, in the context of global environmental governance, social benefits, including resource conservation and carbon emission reduction, have become even more important than pure economic considerations.^92^

Advancing clean production and the circular economy is now a global goal. Source reduction and waste recycling are crucial for transitioning the traditional aluminum industry toward green and sustainable practices. In view of the above, specific recommendations for carbon dross–pollution control are proposed (Table 1), providing insights for low-carbon and high-value development in the aluminum sector.

**Table 1:** Suggestions for pollution prevention and control of carbon dross

Principles	Clean	Cycle	Control
Management	● Reinforced management of daily inspection operations	■ Amendment and revision of laws	◆ Multipronged approach and strict regulation
	● Regular training	■ Issuance of new administrative regulations	◆ Disposal charges enhanced
	● Worker-quality improvement	■ Government introduction of subsidies, demonstrations and promotion	
	● Standardised management of electrolytic production operations	■ Provinces and cities introduce policies related to carbon dross and hazardous waste	◆ Policy discouraging landfills
	● Standardised management of anode raw materials, production operations and post-treatments	■ Industry promotes the development and implementation of standards and norms	
Technology	● R&D and application of high-quality anode-production technology	■ Development and application of flotation technology with high-efficiency separation	◆ Deep defluoridation/decarbonisation technology for recovery products
	● Electrolysis-process-technology upgrade	■ Development and application of fire refining and decarbonisation technology	
	● Research and development and application of independent carbon dross salvage devices	■ Combined application of flotation–fire processes	
	● R&D and application of electrolysis raw/auxiliary material deep decontamination technology	■ Development and application of intensive technology for product recovery	

## 6. Conclusion

Electrolytic aluminum carbon dross, a hazardous waste rich in fluoride and carbon, is proliferating globally due to the expanding aluminum production, posing both environmental risks and resource opportunities. This review examines carbon dross across its “generation–hazards–prevention–control” chain, elucidating its multi-mechanistic formation *via* anode corrosion, spalling, and entrainment, and highlighting the impacts of fluoride and carbon on ecological and operational systems. A novel 3C (clean-cycle-control) framework is proposed: Clean emphasizes source reduction through inert anodes, energy–efficient processes, and smart optimization; cycle enables resource recovery *via* coupled flotation-pyrometallurgy and synergistic production of high-value carbon materials and aluminum fluoride; Control establishes a policy-technology-management integration supported by cross-scale risk assessment to ensure safe disposal. Future work should prioritize the application of clean production technologies, such as inert anodes, to reduce carbon dross emissions at the source. Additionally, for the generated carbon dross, policies should guide the integration of technology and funding, facilitating the transition from passive landfilling to a low-carbon, high-value resource recycling model, supporting the global green transformation and zero-waste goals.

## Author contributions

Ningning Feng: writing – review & editing, writing – original draft, funding acquisition. Chenquan Wang: writing – original draft. Chunqiang Chen: methodology. Xi Liu: investigation, conceptualization. Qiang Huo: writing – review & editing, supervision, funding acquisition.

## Conflicts of interest

The authors declare that they have no known competing financial interests or personal relationships that could have appeared to influence the work reported in this paper.

## Supplementary Material

RA-015-D5RA04272K-s001

## Data Availability

No primary research results, software or code have been included, and no new data were generated or analysed as part of this review. Supplementary information: the thermodynamic analysis of electrochemical reactions in the aluminum electrolysis cell, together with a comparative analysis of various carbon dross treatment technologies, is provided in the SI. See DOI: https://doi.org/10.1039/d5ra04272k.

## References

[cit1] Ishak R., Laroche G., Lamonier J.-F., Ziegler D. P., Alamdari H. (2017). ACS Sustain. Chem. Eng..

[cit2] Harmaji A., Jafari R., Simard G. (2024). Materials.

[cit3] Huo Q., Li R., Chen M., Zhou R., Li B., Chen C., Liu X., Xiao Z., Qin G., Huang J. (2024). J. Hazard. Mater..

[cit4] Tabereaux A. T. and Peterson R. D., in Treatise on Process Metallurgy, Elsevier, 2024, pp. 625–676

[cit5] International-Aluminium-Institute , Current Statistics, 2024, https://international-aluminium.org

[cit6] Chen X., Zhao L., Luo Z. (2009). Light Met..

[cit7] Zhang Y. , Chai D., Zhou Y., Wang Y. F., Bai W. and Hou G., World Nonferrous Metals, 2018, vol. 7, pp. 1–3

[cit8] Mao S., Zhang Q. (2021). Front. Mater..

[cit9] Peterson S. D. , RCRA's Solid Waste Regulation and its Impact on Resource Recovery in the Minerals Industry, US Department of the Interior, Bureau of Mines, 1990

[cit10] López-Delgado A., Robla J. I., Padilla I., López-Andrés S., Romero M. (2020). J. Clean. Prod..

[cit11] Wang C., Li S., Guo Y., He Y., Liu J., Liu H. (2023). J. Environ. Manage..

[cit12] Huo Q., Wang C., Li R., Liu X. (2023). Carbon Technol..

[cit13] Yang J.-S., Xie Y.-J., He W. (2011). Carbohydr. Polym..

[cit14] Zhou L., Yao Z., Sun K., Tian Z., Li J., Zhong Q. (2024). Energies.

[cit15] Patharabe R. R. , Design and Modeling of an Aluminum Smelting Process to Analyze Aluminum Smelter and Identify the Alternative Uses of Nuclear Power Small Modular Reactor, Missouri University of Science and Technology, 2017

[cit16] Chevarin F., Lemieux L., Picard D., Ziegler D., Fafard M., Alamdari H. (2015). Fuel.

[cit17] Lai Y., Liu Y. (2002). Light Met..

[cit18] Tu X. (2017). Shanxi Metall..

[cit19] Xu H., Fu C., Xu R. (2015). China Environ. Monit..

[cit20] Min-Zhang L., Xian L. I. (2012). Carbon Tech..

[cit21] Li Y., Zhang H., Zhang Z., Shao L., He P. (2015). J. Environ. Sci..

[cit22] Liu C. and Wu S., World Nonferrous Metals, 2020, vol. 14, pp. 19–23

[cit23] Zhang Y. , Energy Conservation in Nonferrous Metallurgy, 2021, vol. 37, pp. 21–26

[cit24] Allard F., Désilets M., Blais A. (2019). Thermochim. Acta.

[cit25] Chae Y., Kim D., An Y.-J. (2018). Ecotoxicol. Environ. Saf..

[cit26] Li G., Zheng X., Zhu Y., Long Y., Xia X. (2022). Sci. Total Environ..

[cit27] He L., Tu C., He S., Long J., Sun Y., Sun Y., Lin C. (2021). Environ. Geochem. Health.

[cit28] Tang Y., Ma L., Qiu Z., Yang W., Chen B., Lin Y. (2024). Process Saf. Environ. Prot..

[cit29] Makoba E. (2021). J. Afr. Earth Sci..

[cit30] Yang F., Yu Q., Zuo Z., Hou L. (2021). Energy Sources, Part A.

[cit31] Zhang X. (2021). Light Met..

[cit32] Fellner P., Hiveš J., Korenko M., Thonstad J. (2001). Electrochim. Acta.

[cit33] Shakiba N., Khoei S. M. M. (2021). Ceram. Int..

[cit34] Zhou H. (2013). Light Met..

[cit35] Dellen J., Lynen L., Schwedt A., Mayer J., Telle R. (2020). Tribol. Int..

[cit36] Chen G., Li Y., Huang L., Peng J., Tang L., Luo X. (2022). J. Hazard. Mater..

[cit37] Parshin S. G., Levchenko A. M., Wang P. (2021). Materials.

[cit38] Li H., Wang J., Hou W., Li M., Cheng B., Feng Y., Xu T. (2021). Metals.

[cit39] Chen X., Zhao L., Luo Z. (2009). Light Met..

[cit40] Kang Z., Li S., Lian Y., Zhang L., Yang H. (2022). Chin. Nonferrous Metals.

[cit41] Zhou J., Wu C., Zhang J., Wu Q. (2014). Nonferrous Met..

[cit42] Liu Y., Hu G., Sun W., Zhang Y., Wang L. (2021). Miner. Resour. Util..

[cit43] Chai D., Hou G., Huang H. (2016). Light Met..

[cit44] Wang H., Feng Q., Tang X., Liu K. (2016). Sep. Sci. Technol..

[cit45] Yang K., Gong P., Xin X., Tian Z., Lai Y. (2020). J. Taiwan Inst. Chem. Eng..

[cit46] Zhao Q., Wang Y., Dong H., Wang J., Fu X., Cui X., Li S., Li C. (2021). J. Environ. Chem. Eng..

[cit47] Tian Z., Gong P., Xin X., Yang K., Deng C., Yin K. (2021). Miner. Eng..

[cit48] Yang K., Li J., Huang W., Zhu C., Tian Z., Zhu X., Fang Z. (2022). J. Environ. Manage..

[cit49] Kondrat'ev V., Rzhechitskii E., Shakhrai S., Karlina A., Sysoev I. (2016). Metallurgist.

[cit50] Ma L., Yang W., Cui Y., Chen B., Jiang J., Lin Y. (2023). J. Clean. Prod..

[cit51] He L., Wang X., Zhou J., Li B., Peng Q., Yao Z., Liu W. (2024). JOM.

[cit52] Zhu X., Mao Q., Zhong Q., Zhang Z., Wang G., Tang L., Xiao J. (2022). ACS Omega.

[cit53] Sánchez-Hernández R., Padilla I., López-Andrés S., López-Delgado A. (2017). J. Clean. Prod..

[cit54] Wu S., Tao W., Ge H., Yang J., Chen L., He J., Yang Y., Wang Z. (2023). Sep. Purif. Technol..

[cit55] Souza M. T., Simão L., Montedo O. R. K., Pereira F. R., de Oliveira A. P. N. (2019). Waste Manage..

[cit56] Lipowska B., Kusiorowski R., Gerle A., Śliwa A. (2022). Cleaner Waste Systems.

[cit57] Zhu Z., Xu L., Han Z., Liu J., Zhang L., Tian S., Xu Y., Koppala S. (2022). Miner. Eng..

[cit58] Chen K., Hu C. (2017). Light Met..

[cit59] Zhang L. , Energy Conservation in Nonferrous Metallurgy, 2022, vol. 38, pp. 35–39

[cit60] Kubiňáková E., Danielik V., Híveš J. (2018). Electrochim. Acta.

[cit61] Tkacheva O., Arkhipov P., Kataev A., Rudenko A., Zaykov Y. (2021). Electrochem. Commun..

[cit62] Fang Z., Dang Y., Peng J., Han Z., Ma N., Lv X., Liu M., Li L. (2018). Nanosci. Nanotechnol. Lett..

[cit63] Cassayre L., Palau P., Chamelot P., Massot L. (2010). J. Chem. Eng. Data.

[cit64] Lalancette F., Désilets M., Pansiot B., LeBreux M., Bilodeau J.-F. (2023). Int. J. Heat Mass Transfer.

[cit65] Zhang C. , PhD thesis, University of Science and Technology Beijing, 2022

[cit66] Kang N. , Energy Conservation in Nonferrous Metallurgy, 2004, vol. 1, pp. 30–32

[cit67] Wei Y. (2018). Light Met..

[cit68] Zhou L., Tang L. (2021). J. Environ. Manage..

[cit69] Haraldsson J., Johansson M. T. (2018). Renew. Sustain. Energy Rev..

[cit70] Gupta A., Basu B. (2019). Trans. Indian Inst. Met..

[cit71] He Y., Zhou K.-c., Zhang Y., Xiong H.-w., Zhang L. (2021). J. Mater. Chem. A.

[cit72] Zhang L., Liu Z., Qi J., Chen L., Gao N., Li B. (2024). J. Environ. Chem. Eng..

[cit73] Chen D. , Master's thesis, Central South University, 2012

[cit74] Gao Q. , Master's thesis, Northeastern University, 2015

[cit75] Ma J. , PhD thesis, Northeastern University, 2019

[cit76] Xi J. , PhD thesis, Northeastern University, 2006

[cit77] Wang Z. , PhD Dissertation, Central South University, 2009

[cit78] Kang M., Zhao W., Jia L., Liu Y. (2020). Chin. Geogr. Sci..

[cit79] Zhou B., Wang T., Xu T., Li C., Li J., Fu J., Zhang Z., Song Z., Ma C. (2021). Fuel.

[cit80] Shi M., Xin Y., Chen X., Zou K., Jing W., Sun J., Chen Y., Liu Y. (2021). J. Alloys Compd..

[cit81] Rashidi N. A., Chai Y. H., Ismail I. S., Othman M. F. H., Yusup S. (2022). Biomass Convers. Biorefin..

[cit82] González D., Marí A., Baeza J., Calvo L., Gilarranz M. (2021). J. Environ. Chem. Eng..

[cit83] Jiménez C., Cerrillo M. I., Martínez F., Camarillo R., Rincón J. (2020). Sep. Purif. Technol..

[cit84] Licona-Aguilar Á. I., Lois-Correa J. A., Torres-Huerta A. M., Domínguez-Crespo M. A., Dorantes-Rosales H. J., García-Zaleta D. S. (2020). J. Nanosci. Nanotechnol..

[cit85] Han Z., Kong S., Cheng J., Sui H., Li X., Zhang Z., He L. (2019). Ind. Eng. Chem. Res..

[cit86] An Y., Li C., Sun X., Wang K., Su F., Liu F., Zhang X., Ma Y. (2021). J. Phys. D: Appl. Phys..

[cit87] Dang L., Guo J. K., Kong L. B. (2021). Chemelectrochem.

[cit88] Wu S., Tao W., Zheng Y., Ge H., He J., Yang Y., Wang Z. (2021). Waste Manage..

[cit89] Wu S., Tao W., Zheng Y., Yang Y., Yu J., Cui J., Lu Y., Shi Z., Wang Z. (2020). Hydrometallurgy.

[cit90] Li N. , Gao L. and Chattopadhyay K., in Light Metals 2019, Springer, 2019, pp. 867–872

[cit91] Chai D., Hou G., Huang H. (2016). Light Met..

[cit92] Tsang A., Frost T., Cao H. (2023). Br. Account. Rev..

