# Supplementary material for: Researches and applications of pollution prevention and control technologies for carbon dross from electrolytic aluminium production: a review
Source: RSC Adv. 2025 Oct 16;15(46):38698–713. doi: 10.1039/d5ra04272k (PMC12529064; doi:10.1039/d5ra04272k)
Supplement: RA-015-D5RA04272K-s001 [file RA-015-D5RA04272K-s001.pdf]

Supplementary Information (SI) for RSC Advances.
This journal is © The Royal Society of Chemistry 2025

# Supplementary Information

## Researches and applications of pollution prevention and control technologies for carbon dross from electrolytic aluminium production: a review

Ningning Feng[a, †], Chenquan Wang[b, c, †], Chunqiang Chen[b], Xi Liu[b] and Qiang Huo[b, *]

[a] *School of Life Science and Technology, Inner Mongolia University of Science & Technology, Baotou 014010, China*

[b] *Key Laboratory of Ecology of Rare and Endangered Species and Environmental Protection (Guangxi Normal University), Ministry of Education, Guangxi Guilin, 541006, China*

[c] *Jiading District Environmental Monitoring Station, Shanghai, 201822, China*

**Text S1: Thermodynamic analysis of electrochemical reactions in the aluminum electrolysis cell**

Modeling Principles & Approach:

1. Ionization effects of the chemical compounds and electrolytes were not considered.
2. Aluminum oxide and carbon are considered to be dissolved in the electrolyte at the initial ambient conditions.
3. Dissociation rate of $Al_2O_3$ was kinetically dependent on activation energy and the concentration.
4. Electrochemical deposition potential of $Al_2O_3$ electrolyzed with carbon anodes was considered to be known, $E_d$ = (-1.233/2) = -0.6115 V at 967°C.
5. Dynamic electrical heating was provided to the electrolytic reduction pot.
6. Reaction temperature, pressure and volume variables were dynamic.
7. Equilibrium constant was varying with respect to temperature.
8. Initial condition for Al Smelter were ambient (i.e. 1 atm pressure, and 25°C temp).

The Heat of reaction was calculated by the equation:

* Corresponding authors at: Key Laboratory of Ecology of Rare and Endangered Species and Environmental Protection (Guangxi Normal University), Ministry of Education, Guangxi Guilin, 541006, China.
*E-mail addresses:* huoqiang@gxnu.edu.cn (Q. Huo).
† The authors contributed equally to this paper.

$$\Delta H^0_{(reaction)} = \Delta H^0_{(product)} - \Delta H^0_{(reactant)} \qquad (1)$$

$$= \left[\left(4\ \Delta H^0_{(Al)}\right) + \left(3\ \Delta H^0_{(CO_2)}\right)\right] - \left[\left(2\ \Delta H^0_{(Al_2O_3)}\right) + \left(3\ \Delta H^0_{(C)}\right)\right]$$

$$= [(4 * 0) + (3 * -394.838)] - [(2 * -1692.437) + (3 * 0)]$$

$$= +2200.36 \text{ kJ/mol}$$

As $\Delta H$ of the reaction was positive, it means that reaction is endothermic and heat is absorbed by the system. The Gibbs free energy for the electrochemical reaction of alumina with carbon anodes in cryolite electrolyte is,

$$\Delta G^0_{(cell)} = -nFE^0_{(cell)} \qquad (2)$$

Where, $E^0_{(cell)}$ = Standard electrode potential at 25°C and 1 atm

n = Number of moles of electrons per mol of products

F = Faraday's constants = 96485 Coulombs/mol

The larger the value of the standard reduction potentials ($E^0$), the easier it is for the element to be reduced (accept electrons). In other words, they are better oxidizing agents. Hence, the Gibb's free energy of formation at 967°C is

$$\Delta G^0_{(cell)} = \Delta G^0_{f(product)} - \Delta G^0_{f(reactant)} \qquad (3)$$

$$= \left[\left(4\ \Delta G^0_{f(Al)}\right) + \left(3\ \Delta G^0_{f(CO_2)}\right)\right] - \left[\left(2\ \Delta G^0_{f(Al_2O_3)}\right) + \left(3\ \Delta G^0_{f(C)}\right)\right]$$

$$= [(4 * 0) + (3 * -396.098)] - [(2 * -1282.255) + (3 * 0)]$$

$$= +1376.216 \text{ kJ/mol}$$

The Gibb's free energies of formation of aluminum and carbon components are zero because they are pure elements and free energies of $CO_2$ and $Al_2O_3$ are taken from JANAF data table. As $\Delta G$ of the electrolytic reduction cell is positive, it means forward reaction is non-spontaneous. The electrochemical reaction of $Al_2O_3$ electrolyzed with carbon anode in cryolite is given by

$$E^0_{(cell)} = \left(-\Delta G^0_{(cell)} / nF\right) \quad (4)$$

$$= (-1{,}376{,}216 \text{ J/mol}) / ((4 * 3) * 96485 \text{ J/gm.eq.volt})$$

$$= -1.189 \text{ V}$$

Where n = Number of electrons per mole of products i.e. $4\,Al^{3+}_{(1iq)} = (4 * 3) = 12$

F = Faraday's constant = 96485 Coulombs/mol

The aluminum, carbon and $CO_2$ are nearly in the pure phase i.e. standard state, but $Al_2O_3$ is in standard phase only when it is at saturation. The Nernst Equation in the form of electro-chemical deposition potential ($E_d$), to calculate equilibrium constant ($K_{eq}$).

$$\Delta G = \Delta G^0 + RT * \ln K_{eq} \quad (5)$$

$$-nFE_d = -nFE^0 + RT * \ln K_{eq} \quad (6)$$

$$E_d = E^0 - \left(\frac{RT}{nF}\right) * \ln\frac{[Oxd]}{[Red]} = E^0 + \left(\frac{RT}{nF}\right) * \ln\frac{[Red]}{[Oxd]} \quad (7)$$

$$E_d = E^0 + \left(\frac{RT}{nF}\right) * \ln K_{eq} \quad (8)$$

$$-0.6115 = -1.189 + \left(\frac{8.314 * T}{12 * 96485}\right) * \ln K_{eq}$$

$$\ln K_{eq} = -2367.445 * \left(\frac{1}{T}\right) \quad (9)$$

This is an equation of Equilibrium constant with respect to varying temperature. In this, $\Delta G^0$ = Standard Gibb's free energy of formation of the cell and $\Delta G$ = Gibb's free energy of formation for $Al_2O_3$ electrolyzed with Carbon Anode. Comparing this with standard Mimic phase equilibrium constant equation,

$$\ln K_{eq} = A_1 + \left(\frac{A_2}{T}\right) + \left(A_3 * \ln(T)\right) + \left(A_4 * T\right) \quad (10)$$

It gives $A_1 = 0$, $A_2 = -2367.445$, $A_3 = 0$ and $A_4 = 0$.

Table S1. Comparative analysis of various carbon dross treatment technologies.

| Technology | Removal/recovery efficiency | Estimated operating cost (RMB/ton) | Energy consumption | Technology maturity&scalability | Main environmental impacts&risks |
|---|---|---|---|---|---|
| Flotation | Both carbon and fluoride recoverable; Carbon recovery rate: 80–90%; Fluoride removal rate: 60–80% | 300-500 | Low | Limited industrial application; scalability currently constrained due to limited product purity | Generates fluoride-containing wastewater requiring further treatment; risk of fluoride contamination in carbon products |
| Roasting | Fluoride recovery rate > 95%; High removal of carbon and fluoride | 1000-2000 | High | Pilot-scale at lab level; scalability limited under emission reduction policies | Carbon not recovered; emits fluoride-containing exhaust gas and dust, requires high-efficiency purification facilities |
| Leaching | High carbon recovery rate; Low fluoride recovery rate | 500-800 | Medium | Laboratory pilot stage; scalability limited due to declining carbon content in carbon dross | Produces acidic/alkaline high-fluoride wastewater; difficult fluoride recovery; generates high-salinity waste brine; increased reagent use raises cost over time |
| Alkali fusion | Effective carbon recovery; High cost for secondary fluoride recovery | 1500–2500 | High | Laboratory R&D phase; challenges in scaling up due to corrosion and energy consumption; limited by decreasing carbon content in slag | High energy use; high fluoride recovery cost; produces alkaline residues and saline wastewater |
| Vacuum smelting | Both carbon and fluoride recoverable; low product purity | ＞4000 | Very high | Laboratory research phase; heavily constrained by equipment and cost | Demands advanced equipment; high energy use; high operation and maintenance costs |
| Landfilling | Not designed for recovery | 800 | Low | Industrially applied; significantly affects maximum allowable waste generation for producers | Long-term fluoride leaching risk; no resource recovery; occupies land resources |
